# Single crystal growth and structure analysis of type-I (Na/Sr)–(Ga/Si) quaternary clathrates†

**DOI:** 10.1039/c9ra01489f

**Published:** 2019-05-09

**Authors:** Hironao Urushiyama, Haruhiko Morito, Hisanori Yamane

**Affiliations:** Institute of Multidisciplinary Research for Advanced Materials, Tohoku University 2-1-1 Katahira, Aoba-ku Sendai 980-8577 Japan; Department of Metallurgy, Materials Science and Materials Processing, Graduate School of Engineering, Tohoku University 6-6-04 Aramaki Aza Aoba, Aoba-ku Sendai 980-8579 Japan; Institute for Materials Research, Tohoku University 2-1-1 Katahira, Aoba-ku Sendai 980-8577 Japan morito@imr.tohoku.ac.jp

## Abstract

Single crystals of (Na/Sr)–(Ga/Si) quaternary type-I clathrates, Na_8−*y*_Sr_*y*_Ga_*x*_Si_46−*x*_, were synthesized by evaporating Na from a mixture of Na–Sr–Ga–Si–Sn in a 6 : 0.5 : 1 : 2 : 1 molar ratio at 773 K for 12 h in an Ar atmosphere. Electron-probe microanalysis and single-crystal X-ray diffraction revealed that three crystals from the same product were Na_8−*y*_Sr_*y*_Ga_*x*_Si_46−*x*_ with *x* and *y* values of 7.6, 2.96; 8.4, 3.80; and 9.1, 4.08. It was also shown that increasing the Sr and Ga contents increased the electrical resistivity of the crystal from 0.34 to 1.05 mΩ cm at 300 K.

## Abstract 2

Single crystals of (Na/Sr)–(Ga/Si) quaternary type-I clathrates, Na_8−*y*_Sr_*y*_Ga_*x*_Si_46−*x*_, were synthesized by evaporating Na from a mixture of Na–Sr–Ga–Si–Sn in a 6 : 0.5 : 1 : 2 : 1 molar ratio at 773 K for 12 h in an Ar atmosphere.

## Graphical abstract

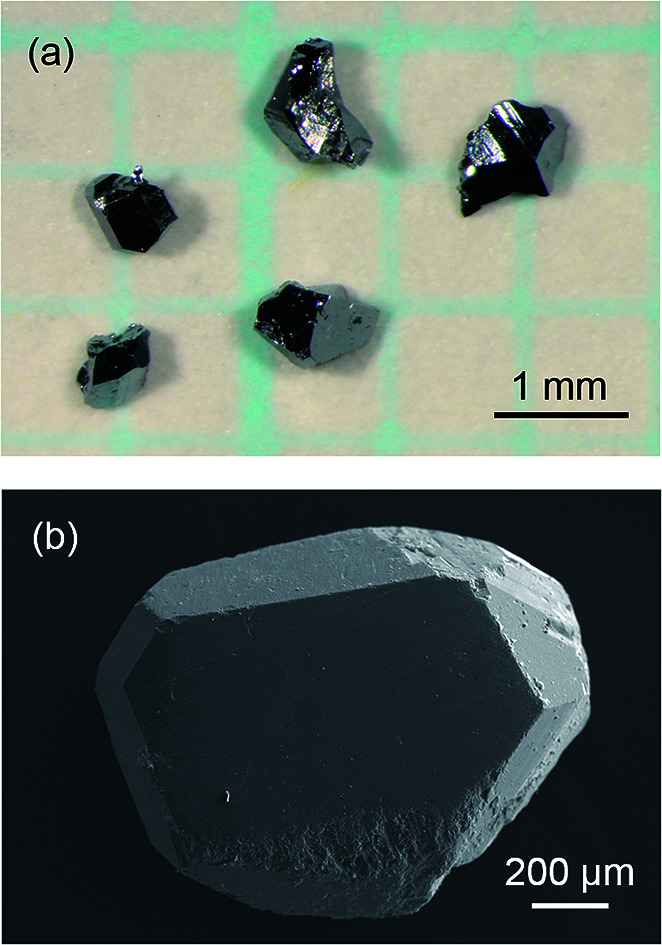

## 1. Introduction

Silicon (Si) clathrate compounds are composed of host Si atoms organized in three-dimensional frameworks and guest atoms enclosed in the Si cages of the frameworks. Kasper *et al.* first synthesized binary Si clathrate, Na_8_Si_46_, in 1965.^1^ Since then, many researchers have studied Si clathrate compounds,^2^ altering their physical properties by partial or full substitution of different elements for the host and guest atoms. Kawaji *et al.* synthesized a type-I clathrate, (Na,Ba)_8_Si_46_;^3^ this compound was the first Si clathrate superconductor with a *T*_C_ value of 4 K derived from the partial substitution of Ba for Na in the Na_8_Si_46_ cages. Another Si clathrate, Ba_8_Si_46_, which was synthesized at high pressure (3 GPa) and 1073 K, exhibited the highest *T*_C_ value 8 K among the various Si clathrate compounds.^4^ The framework Si atoms can also be partially replaced by Ga atoms in some type-I clathrate compounds, such as A_8_Ga_8_Si_38_ (A = K, Rb, Cs), as described by Sui *et al.*^5^ A_8_Ga_8_Si_38_ powder was sintered by spark plasma sintering to obtain bulk polycrystalline samples, which exhibited band gaps in the range of 1.14–1.18 eV.^5^ Another clathrate composed of Ga/Si cages and Ba atoms, Ba_7.94_Ga_15.33_Si_30.67_, was shown to have a relatively high thermoelectric dimensionless figure of merit, *ZT*, of 0.87 at 870 K.^6,7^ Sr_8_Ga_11_Si_35_ ^8^ and Sr_8_Ga_13.6_Si_32.4_,^9^ which contain Sr guest atoms in Ga/Si cages, exhibit electrical resistivities of approximately 0.2 and 0.26 mΩ cm, respectively, at 280 K.

Some ternary silicon clathrates could be synthesized *via* solid-state reactions between each elements at high temperature^5^ or melting method.^6,8,9^ However, the silicon clathrates containing a Na atom could not be synthesized by simple reaction because these clathrates have been regarded as metastable or intermediate phases. These clathrates were generally prepared by thermal decomposition of the precursor compounds. For example, the binary silicon clathrates containing Na, Na_8_Si_46_ and Na_24_Si_136_, were synthesized by thermal decomposition of Na_4_Si_4_ Zintl compound.^1,2^ The clathrate samples obtained by this method were powdery due to the solid state of the precursor compounds. Therefore, it is difficult to prepare the bulk crystal of the silicon clathrates containing Na. In our previous study, the single crystals of the Na–Si binary clathrate were successfully grown by using Sn flux.^10,11^ Single crystals of type-I Na_8_Si_46_ and type-II Na_24_Si_136_ clathrates were selectively grown in Na–Sn rich Na–Sn–Si ternary melt by Na evaporation.^11^ Single crystals of a ternary type-I clathrate, Na_8_Ga_5.70_Si_40.30_, could also be prepared by a self-flux method using Ga as a flux.^12^ Furthermore, the crystal growth of Na_8_Ga_*x*_Si_46−*x*_ (*x* = 4.94–5.52) clathrates was achieved by adding a Sn flux to the starting melt. We could measure the electrical resistivity of the single crystals for these clathrates containing Na. The clathrates exhibited metallic conduction, and their electrical resistivity decreased as the Ga content decreased (*e.g.*, the resistivities of Na_8_Ga_5.70_Si_40.30_ and Na_8_Ga_4.94_Si_41.06_ were 1.40 and 0.72 mΩ cm, respectively, at 300 K).

To extend the variation of clathrate compounds and their field of properties and applications, doping or partial substitution of other atoms at the Na atom site is also designed. Recently, quaternary Ga/Si and Zn/Si clathrates having Na and Rb or Cs guest atoms, such as Cs_6_Na_2_Ga_8.25_Si_37.75_, Rb_6.34_Na_1.66_Ga_8.02_Si_37.98_, and Rb_8_Na_16_Zn_8.4_Si_127.6_, have been synthesized using a Ga or Zn flux.^13^ However, the typical size of the single crystals was below 0.1 mm, and the properties of the crystals could not be characterized. So, synthesis of quaternary Na and Si based clathrate single crystals with a size enough for characterization is still challenging. In the present study, we succeeded in growing the single crystals of quaternary Ga–Si cage clathrate compounds encapsulating Na and Sr guest atoms by the Sn flux method. The compounds are the first examples of the Ga/Si clathrates containing Na (1+) and other guest cations with a different formal ionic charge (2+). The crystal structures and electrical properties were investigated for the single crystals of the new clathrates.

## 2. Experimental methods

The experiments were conducted as described in the previous studies.^10–12^ Metal Na pieces (Nippon Soda Co. Ltd., 99.95%), Si powder (Kojundo Chemical Laboratory Co. Ltd., 4N), Ga grains (Dowa Electronics Co. Ltd., 6N), and Sn granules (Mitsuwa Chemicals Co. Ltd., 5N) were combined by weight at a Na : Ga : Si : Sn molar ratio of 6 : 1 : 2 : 1 (total 8.70 mmol) in a glove box with an Ar atmosphere. The raw material mixture was then put in a boron nitride (BN) crucible (Showa Denko KK; inner diameter of 6.5 mm and depth of 18 mm) and sealed in a stainless steel (SUS) container (SUS316, outer diameter of 12.7 mm, inner diameter of 10.7 mm, and height of 80 mm) with Ar gas. The SUS container was heated in an electric furnace at 1173 K for 12 h then the furnace was cooled to room temperature. The BN crucible was then taken from the SUS container in the glove box and Sr grains (Alfa Aeser, 4N) were added to the Na–Ga–Si–Sn mixture in the BN crucible to make the Na : Sr : Ga : Si : Sn molar ratio 6 : 0.5 : 1 : 2 : 1. Next, the BN crucible was sealed in the upper part of another long SUS container (outer diameter of 12.7 mm, inner diameter of 10.7 mm, and height of 300 mm) with Ar gas. The upper part of the container was heated at 773 K for 12 h, and the lower part was cooled using a fan to keep the temperature almost the same with the room temperature. By generating a temperature gradient in the container, the Na was evaporated from the mixture in the crucible, and condensed on the inner wall in the lower cooler part of the container.

After heating, the crucible was taken out in the glove box, and the weight loss from the sample was measured to calculate the amount of evaporated Na against the amount of Na in the starting mixture. The sample in the crucible was subjected to an alcohol treatment by which any excess Na and Na–Sn and Na–Ga compounds in the sample were completely reacted with 2-propanol followed by ethanol, and the reaction products of Na were removed from the samples by washing with water. A mixture of Ga and Sn remained after the decomposition of Na–Sn and Na–Ga compounds by the alcohol treatment and a Sr–Ga–Si ternary compound in the sample were then subjected to a hydrochloric acid treatment by dissolving in an aqueous hydrochloric acid (35.0–37.0 mass% HCl) and rinsing the residue with water.

The morphologies of the obtained single crystals were observed with an optical microscope (Olympus, SZX16) and a scanning electron microscope (SEM; JEOL, JXA-8200) at an accelerating voltage of 15 kV. The single crystals were cut to about 100–150 μm in size and subjected to X-ray diffraction (XRD) measurements (Bruker, D8 QUEST). APEX3 ^14^ was used to collect the diffraction data and refine the unit cells. X-ray absorption correction was performed by SADABS installed in APEX3.^14^ SHELEXL-97 software^15^ was used to refine the occupancies, coordinates, and displacement parameters of the atoms. The crystal structure was drawn by VESTA.^16^ The compositions of the obtained single crystals were analyzed with an electron-probe microanalyzer (EPMA, JEOL, JXA-8200). The electrical characteristics of the single crystals were measured in the range of 8–300 K by the four-terminal method using Ag paste as electrodes.

## 3. Results and discussion

**Fig. 1:**
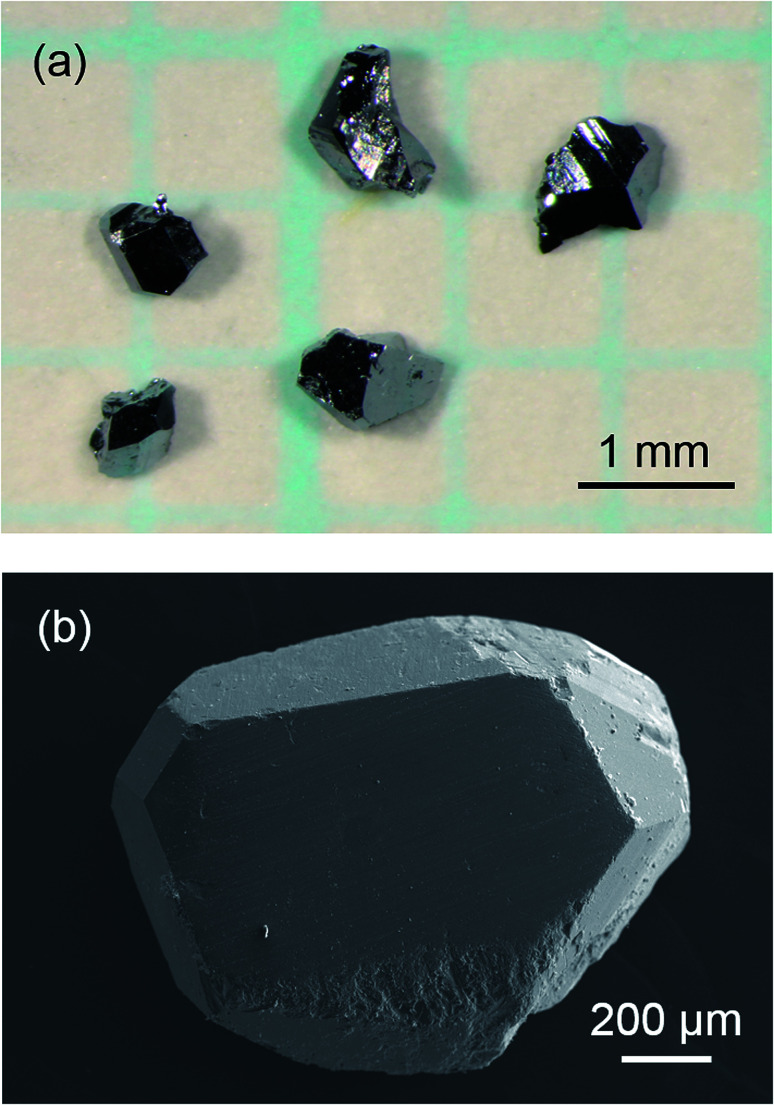
Optical micrograph of the sample after the hydrochloric acid treatment (a) and an SEM image of a Na–Sr–Ga–Si quaternary type-I clathrate single crystal (b).

**Fig. 2:**
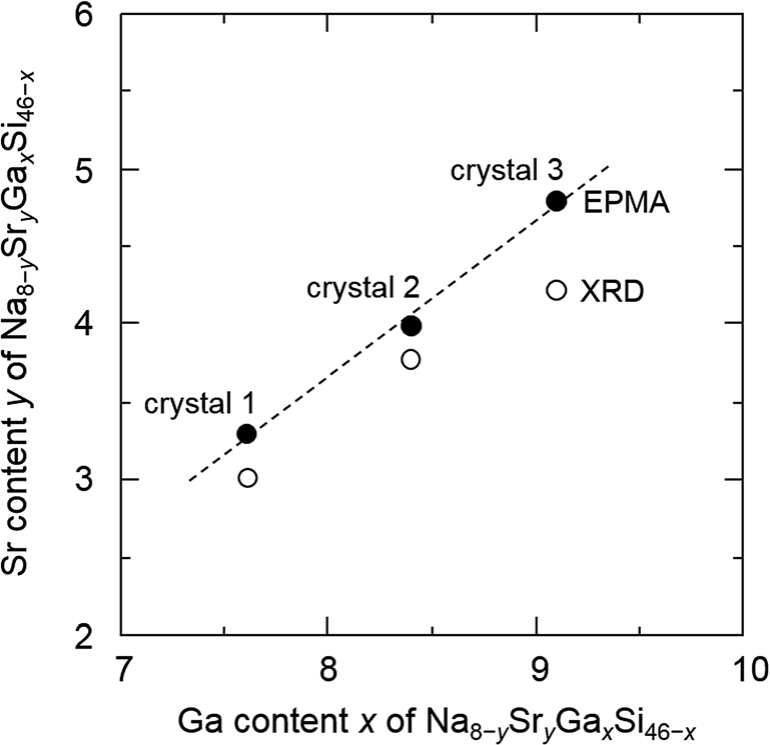
Sr content (*y*) *versus* Ga content (*x*) of Na_8−*y*_Sr_*y*_Ga_*x*_Si_46−*x*_.

When Sr was heated with other starting materials, at 1173 K for 12 h, a SrGaSi ternary compound was crystallized.^17^ Once this compound was formed, it did not melt or dissolve into a liquid phase at 773 K and Sr was not provided to the crystal growth of clathrate. Thus, Sr was added to the Na–Ga–Si–Sn mixture prepared in advance. By heating the Na–Ga–Si–Sn mixture and Sr at 773 K for 12 h, 46% of Na was evaporated. The residual excess Na and Na of Na–Sn and Na–Ga compounds in the sample were removed by the alcohol treatment. After hydrochloric acid treatment for removal of Sn and Ga by decomposition of the Na–Sn and Na–Ga compounds and a SrGaSi compound contained in the product, the black single crystals of clathrate were clearly separated. Fig. 1 shows optical and SEM micrographs of the crystals picked up from the obtained sample. Quantitative EPMA analyses were performed on the flat surfaces of the three black single crystals with sizes of 0.96 mm (crystal 1), 0.93 mm (crystal 2), and 0.83 mm (crystal 3) which were taken from the same sample. The Na, Sr, Ga, and Si contents of crystals 1, 2, and 3 are summarized in Table 1. The chemical formulas of crystals 1, 2, and 3 were calculated by setting the total number of Si and Ga atoms to 46 (based on the general formula of the type-I clathrate, Na_8−*y*_Sr_*y*_Ga_*x*_Si_46−*x*_) as Na_4.9(2)_Sr_3.3(2)_Ga_7.6(2)_Si_38.4(2)_, Na_3.8(5)_Sr_4.0(3)_Ga_8.4(2)_Si_37.3(2)_, and Na_3.2(1)_Sr_4.8(1)_Ga_9.1(1)_Si_36.9(2)_, respectively. The sum of the Na and Sr numbers was close to 8. As shown in Fig. 2, the Sr content, *y*, linearly increased as the Ga content, *x*, increased. The largest crystal, crystal 1, had the smallest Sr and Ga contents among the three crystals.

**Table 1:** Results of the EPMA analysis for crystals 1, 2, and 3

Crystal	Composition (EPMA)				
	Na (at%)	Sr (at%)	Ga (at%)	Si (at%)	Chemical formual (Ga + Si = 46)
1	9.08(44)	6.06(33)	14.01(45)	70.85(31)	Na_4.9(2)_Sr_3.3(2)_Ga_7.6(2)_Si_38.4(2)_
2	7.03(91)	7.48(47)	15.66(32)	69.83(32)	Na_3.8(5)_Sr_4.0(3)_Ga_8.4(1)_Si_37.6(2)_
3	5.88(3)	8.89(13)	16.93(18)	68.30(27)	Na_3.2(1)_Sr_4.8(1)_Ga_9.1(1)_Si_36.9(2)_

In addition, cross sections of the crystals were also analyzed by EPMA; the results are shown in Fig. S1.† In crystals 2 and 3, Na, Sr, Ga, and Si were homogeneously distributed. In contrast, in crystal 1, the surface was Na_4.9(2)_Sr_3.3(2)_Ga_7.6(2)_Si_38.4(2)_, but its composition changed sharply at one region that did not contain Sr and had a composition of Na_8.3(2)_Ga_4.3(2)_Si_41.7(2)_. The crystal 1 containing the Na–Ga–Si ternary clathrate part which were surrounded with the low Sr content Na_4.9(2)_Sr_3.3(2)_Ga_7.6(2)_Si_38.4(2)_ was probably grown at the early stage of the crystal formation. This may indicate that Sr which was added to the Na–Ga–Si–Sn mixture was gradually provided to the melt during heating at 773 K. Homogeneous and high Sr concentrations in the crystals 2 and 3 suggest the crystal growth from Sr-rich melts at later stages. Further studies are needed to clarify the process by which crystals with different Sr and Ga contents are grown from the same starting mixture.

The results of the X-ray crystal structure analyses of pieces from the surface of crystals 1–3 are listed in Tables 2–4. The crystal structures were analyzed based on the model of a type-I clathrate (space group, *Pm*3̄*n*). The occupancies of Si/Ga1(24k), Si/Ga2(16i), and Si/Ga3(6c) were refined under the constraint that the total Ga content was equal to that measured by EPMA. The reliability factor, *R*1(all), was in the range of 1.13–1.34% for all analyses. The chemical formulas of crystals 1, 2, and 3 were calculated from the refined occupancies as Na_5.04_Sr_2.96(2)_Ga_7.6_Si_38.4_, Na_4.20_Sr_3.80(3)_Ga_8.4_Si_37.6_, and Na_3.92_Sr_4.08(2)_Ga_9.1_Si_36.9_, respectively. The Sr contents were relatively consistent with those derived by EPMA (3.3(2), 4.0(3), 4.8(1)). The unit cell constants increased with the increase of the Ga and Sr contents (10.3645(3), 10.3747(3), and 10.3804(4) Å for crystals 1, 2, and 3, respectively).

**Table 2:** Crystal data, data collection, and refinement for the XRD analysis of Na–Sr–Ga–Si quaternary single crystals^a^

	Crystal 1	Crystal 2	Crystal 3
Chemical formula	Na_5.04_Sr_2.96(2)_Ga_7.6_Si_38.4_	Na_4.20_Sr_3.80(3)_Ga_8.4_Si_37.6_	Na_3.92_Sr_4.08(2)_Ga_9.1_Si_36.9_
Formula weight, *M*_r_ (g mol^−1^), *Z*	1983.75, 1	2071.35, 1	2118.58, 1
Temperature, *T* (K)	302(2)	302(2)	302(2)
Crystal system, space group	Cubic, *Pm*3̄*n*	Cubic, *Pm*3̄*n*	Cubic, *Pm*3̄*n*
Unit-cell dimension, *a* (Å)	10.3645(3)	10.3747(3)	10.3804(4)
Unit-cell volume, *V* (Å^3^)	1113.38(10)	1116.67(10)	1118.52(13)
Calculated density, *D*_cal_ (Mg m^−3^)	2.958(5)	3.080(6)	3.146(6)
Radiation wavelength, *l* (Å)	0.71073	0.71073	0.71073
Size (mm^3^)	0.216 × 0.160 × 0.152	0.141 × 0.174 × 0.183	0.142 × 0.152 × 0.149
Absorption correction	Multi-scan	Multi-scan	Multi-scan
absorption coefficient, *μ* (mm^−1^)	9.139	10.544	11.248
Limiting indices	−11 ≤ *h* ≤ 14	−10 ≤ *h* ≤ 14	−13 ≤ *h* ≤ 9
	−10 ≤ *k* ≤ 13	−12 ≤ *k* ≤ 12	−10 ≤ *k* ≤ 14
	−12 ≤ *l* ≤ 10	−14 ≤ *l* ≤ 11	−11 ≤ *l* ≤ 13
*F*_000_	941	977	997
*θ* range for date colletion (°)	2.779–28.686	2.777–28.656	2.775–28.638
Reflections collected/unique	3300/284	3777/284	2901/284
*R*_int_	0.0246	0.0310	0.0389
Date/restraints/parameters	284/1/21	284/1/21	284/1/21
Weight parameters, *a*, *b*	0.0101, 0.2875	0.0129, 0.2629	0.0101, 0.2209
Goodness-of-fit on *F*^2^, *S*	1.223, 1.220	1.191, 1.189	1.223, 1.220
*R*_1_, w*R*_2_ (*I* > 2*σ*(*I*))	0.0113, 0.0254	0.0113, 0.0283	0.0134, 0.0311
*R*_1_, w*R*_2_ (all date)	0.0124, 0.0258	0.0125, 0.0287	0.0143, 0.0314
Largest diff. park and hole, Δ*ρ* (eÅ^−3^)	0.326, −0.242	0.261, −0.239	0.329, −0.244

a *R*_1_ *=* Σ||*F*_o_| − |*F*_c_||/Σ|*F*_o_|. w*R*_2_ = [Σw(*F*_o_^2^ − *F*_c_^2^)^2^/Σ(w*F*_o_^2^)^2^]^1/2^, w = 1/[*σ*^2^(*F*_o_^2^) + (*aP*)^2^ + *bP*], where *F*_o_ is the observed structure factor, *F*_c_ is the calculated structure factor, *σ* is the standard deviation of *F*_c_^2^, and *P* = (*F*_o_^2^ + 2*F*_c_^2^)/3. *S* = [Σw(*F*_o_^2^ − *F*_c_^2^)^2^/(*n* − *p*)]^1/2^, where *n* is the number of reflections and *p* is the total number of parameters refined.

**Table 3:** Atomic coordinates and equivalent isotropic displacement parameters (*U*_eq_/Å^2^) of crystals 1, 2, and 3

Atom	Site	Occupancy	*x*	*y*	*z*	*U*_eq_
**Crystal 1 (Na**_**5.04**_ **Sr**_**2.96(2)**_ **Ga**_**7.6**_ **Si**_**38.4**_ **)**						
Na/Sr 1	6d	0.709/0.291(3)	1/4	1/2	0	0.0440(4)
Na/Sr 2	2a	0.395/0.605(3)	0	0	0	0.0113(2)
Si/Ga 1	24k	0.8995/0.1005(7)	0	0.30576(3)	0.11653(3)	0.00852(12)
Si/Ga 2	16i	0.9656/0.0344(10)	0.18442(3)	*x*	*x*	0.00811(15)
Si/Ga 3	6c	0.227/0.773(2)	1/4	0	1/2	0.00876(13)

**Crystal 2 (Na**_**4.20**_ **Sr**_**3.80(3)**_ **Ga**_**8.4**_ **Si**_**37.6**_ **)**						
Na/Sr 1	6d	0.600/0.400(3)	1/4	1/2	0	0.0420(4)
Na/Sr 2	2a	0.300/0.700(4)	0	0	0	0.0104(2)
Si/Ga 1	24k	0.8775/0.1225(7)	0	0.30587(3)	0.11661(3)	0.00834(13)
Si/Ga 2	16i	0.9521/0.0479(10)	0.18454(3)	*x*	*x*	0.00800(16)
Si/Ga 3	6c	0.218/0.782(2)	1/4	0	1/2	0.00853(15)

**Crystal 3 (Na**_**3.92**_ **Sr**_**4.08(2)**_ **Ga**_**9.1**_ **Si**_**36.9**_ **)**						
Na/Sr 1	6d	0.565/0.435(3)	1/4	1/2	0	0.0423(4)
Na/Sr 2	2a	0.266/0.734(3)	0	0	0	0.0114(2)
Si/Ga 1	24k	0.8613/0.1387(8)	0	0.30578(4)	0.11662(4)	0.00884(14)
Si/Ga 2	16i	0.9387/0.0613(11)	0.18452(3)	*x*	*x*	0.00851(17)
Si/Ga 3	6c	0.202/0.798(2)	1/4	0	1/2	0.00879(15)

**Table 4:** Anisotropic displacement parameters (*U*_*ij*_/Å^2^) for crystals 1, 2, and 3

Atom	*U*_11_	*U*_22_	*U*_33_	*U*_23_	*U*_13_	*U*_12_
**Crystal 1 (Na**_**5.04**_ **Sr**_**2.96(2)**_ **Ga**_**7.6**_ **Si**_**38.4**_ **)**						
Na/Sr1	0.0272(5)	0.0524(5)	=*U*_11_	0	0	0
Na/Sr2	0.0113(2)	=*U*_11_	=*U*_11_	0	0	0
Si/Ga1	0.00866(18)	0.00866(18)	0.00825(18)	−0.00038(12)	0	0
Si/Ga2	0.00811(15)	=*U*_11_	=*U*_11_	−0.00057(9)	=*U*_23_	=*U*_23_
Si/Ga3	0.0099(2)	0.00819(15)	=*U*_22_	0	0	0

**Crystal 2 (Na**_**4.20**_ **Sr**_**3.80(3)**_ **Ga**_**8.4**_ **Si**_**37.6**_ **)**						
Na/Sr1	0.0244(5)	0.0508(5)	=*U*_11_	0	0	0
Na/Sr2	0.0104(2)	=*U*_11_	=*U*_11_	0	0	0
Si/Ga1	0.00860(18)	0.00827(18)	0.00814(19)	−0.00028(12)	0	0
Si/Ga2	0.00800(16)	=*U*_11_	=*U*_11_	−0.00058(10)	=*U*_23_	=*U*_23_
Si/Ga3	0.0097(2)	0.00819(16)	=*U*_22_	0	0	0

**Crystal 3 (Na**_**3.92**_ **Sr**_**4.08(2)**_ **Ga**_**9.1**_ **Si**_**36.9**_ **)**						
Na/Sr1	0.0244(5)	0.0512(5)	=*U*_11_	0	0	0
Na/Sr2	0.0114(2)	=*U*_11_	=*U*_11_	0	0	0
Si/Ga1	0.0090(2)	0.0089(2)	0.0086(2)	−0.00038(13)	0	0
Si/Ga2	0.00851(17)	=*U*_11_	=*U*_11_	−0.00049(11)	=*U*_23_	=*U*_23_
Si/Ga3	0.0101(2)	0.00815(17)	=*U*_22_	0	0	0

**Fig. 3:**
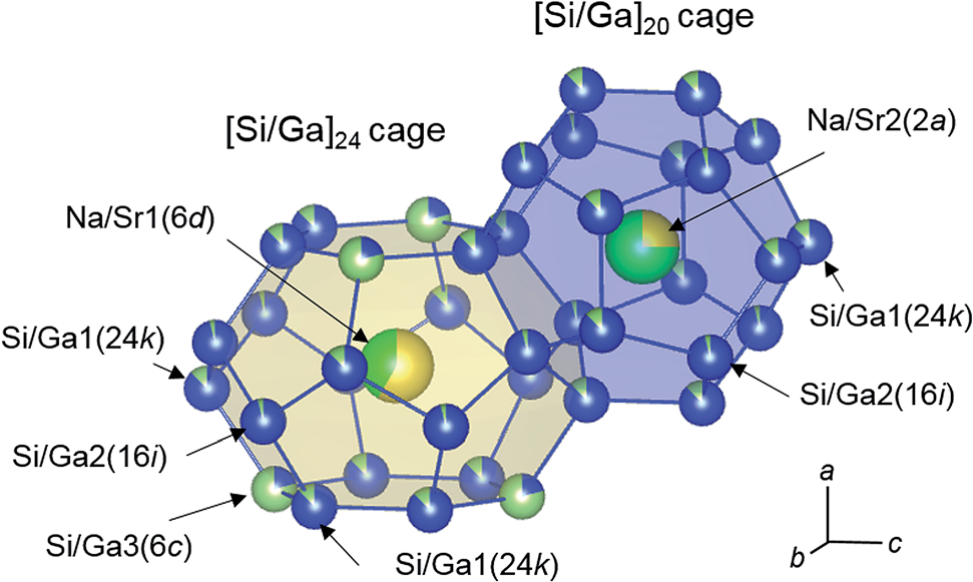
Structures of the [Si/Ga]_20_ and [Si/Ga]_24_ cages of Na_3.9_Sr_4.1_Ga_9.1_Si_36.9_ (Na: yellow, Ga: yellow-green, Si: blue, Sr: green). The occupancies are represented by the surface areas of the spheres.

The pentagonal dodecahedral ([Si/Ga]_20_) and tetrakaidecahedral ([Si/Ga]_24_) cages of Na_3.92_Sr_4.08(2)_Ga_9.1_Si_36.9_ (crystal 3) are depicted in Fig. 3. In crystals 1, 2, and 3, the Ga occupancies for the Si/Ga1(24k), Si/Ga2(16i), and Si/Ga3(6c) sites ranged from 0.1005(7) to 0.1387(8), 0.0344(10) to 0.0613(11), and 0.773(2) to 0.798(2), respectively, and Ga atoms preferentially occupied the Si/Ga3(6c) sites in the [Si/Ga]_24_ cages. Similar preferential occupation of Ga atoms in the Si/Ga3(6c) sites has been previously reported for other type-I clathrates, Rb_6.34_Na_1.66(2)_Ga_8.02_Si_37.98(3)_ and Cs_6_Na_2_Ga_8.25_Si_37.75(3)_.^13^ The occupancies of the Sr atoms for the Na/Sr2(2a) sites in the [Si/Ga]_20_ cages (0.605(3)–0.734(3)) were larger than those for the Na/Sr1(6d) sites in the [Si/Ga]_24_ cages (0.291(3)–0.435(3)). Further, the volumes of the [Si/Ga]_20_ cages (107.2–107.8 Å^3^) were smaller than those of the [Si/Ga]_24_ cages (142.8–143.4 Å^3^). In the Cs_6_Na_2_Ga_8.25_Si_37.75_ and Rb_6.34_Na_1.66_Ga_8.02_Si_37.98_ crystals, Cs and Rb fully occupy the 6d sites inside the larger cage of [Si/Ga]_24_, which indicates selective occupation of larger cations in larger cages.^13^ In the present study, Sr atom preferred to occupy the 2a sites in the smaller [Si/Ga]_20_ cage despite the atomic size of Sr larger than that of Na. This result suggests that the cation size is not related to the site preference in the Na_8−*y*_Sr_*y*_Ga_*x*_Si_46−*x*_ clathrate. Since the electronegativities (Pauling scale^18^) of Rb (*χ*^Rb^_P_ = 0.82) and Cs (*χ*^Cs^_P_ = 0.79) in the Cs_6_Na_2_Ga_8.25_Si_37.75_ and Rb_6.34_Na_1.66_Ga_8.02_Si_37.98_ were smaller than that of Na (*χ*^Na^_P_ = 0.93), the outermost electrons of Rb and Cs atoms could effectively be transferred to the more electronegative Ga atoms (*χ*^Ga^_P_ = 1.81). In the case of Na_8−*y*_Sr_*y*_Ga_*x*_Si_46−*x*_, the electronegativities of Sr (*χ*^Sr^_P_ = 0.95) and Na (*χ*^Na^_P_ = 0.93) were similar, but the first ionization energy of Na (5.139 eV) was smaller than that of Sr (5.695 eV).^19^ Thus, Na atoms may preferentially occupy the Ga-rich [Si/Ga]_24_ cages of Na_8−*y*_Sr_*y*_Ga_*x*_Si_46−*x*_ clathrates.

The crystal structure of Na_3.92_Sr_4.08(2)_Ga_9.1_Si_36.9_ (crystal 3) is drawn with displacement ellipsoids representing the 99% probability region in Fig. S2.† The *U*_22_ = *U*_33_ parameters of the Na/Sr1(6d) sites in crystals 1, 2, and 3 were 0.0508(4), 0.0524(5), and 0.0512(5) Å^2^, respectively, which were larger than the atomic displacement parameters of *U*_11_ (0.0272(5), 0.0244(5), and 0.0244(5) Å^2^) and much larger than those of *U*_*ij*_ at other sites (≤0.0114(2) Å^2^) (Table 4). This probably corresponds to the large, distorted shape of the [Si/Ga]_24_ cages. Such large anisotropic atomic displacement parameters of *U*_22_ = *U*_33_ for the 6d sites in [Si/Ga]_24_ cages have similarly been reported for other type-I clathrates including Na_8_Ga_*x*_Si_46−*x*_ (4.94(6) ≤ *x* ≤ 5.70(7)),^12^ A_8_Ga_8_Si_38_ (A = K, Rb, Cs),^5^ Rb_6.34_Na_1.66(2)_Ga_8.02_Si_37.98(3)_, and Cs_6_Na_2_Ga_8.25_Si_37.75(3)_.^13^

**Fig. 4:**
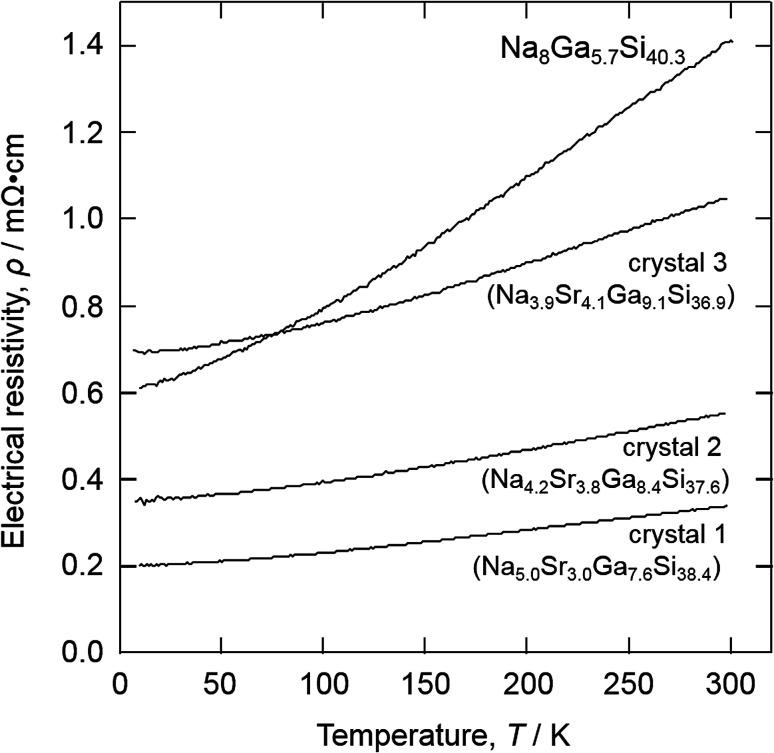
Temperature dependence of the electrical resistivity of crystal 1 (Na_5.0_Sr_3.0_Ga_7.6_Si_38.4_), crystal 2 (Na_4.2_Sr_3.8_Ga_8.4_Si_37.6_), crystal 3 (Na_3.9_Sr_4.1_Ga_9.1_Si_36.9_), and Na_8_Ga_5.7_Si_40.3_.^12^

**Fig. 5:**
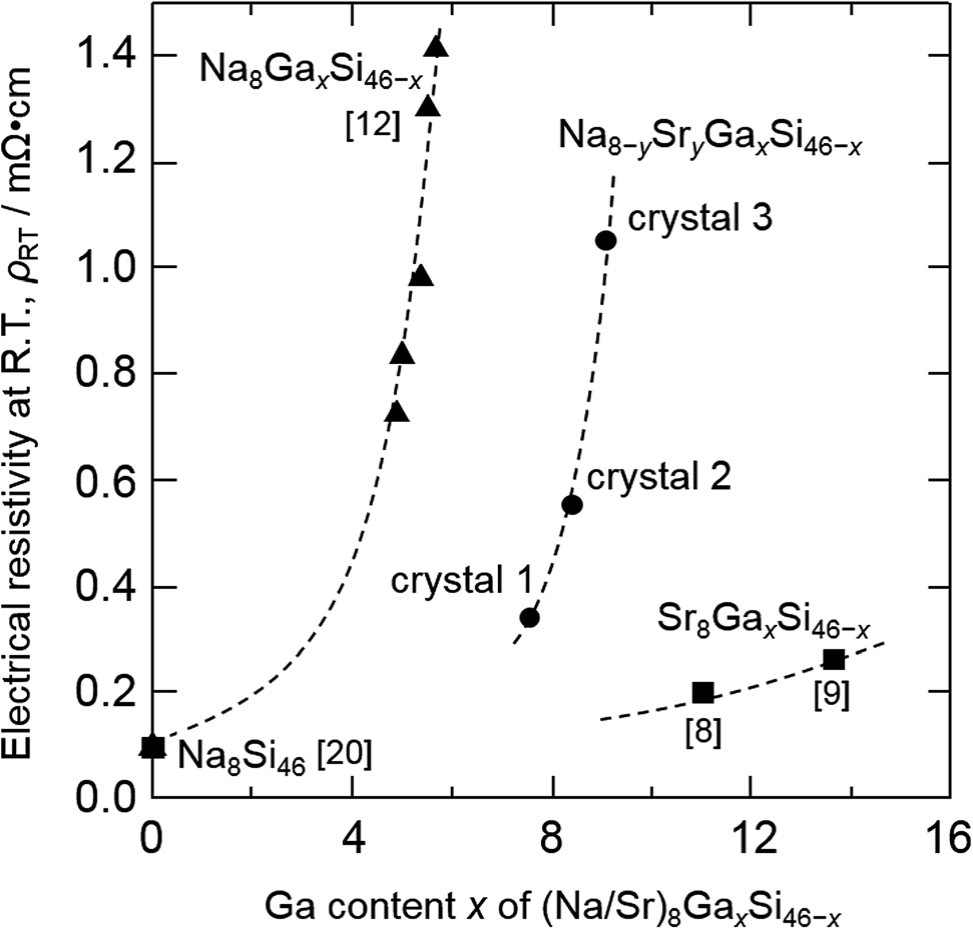
Electrical resistivity at room temperature *versus* Ga content of type-I clathrates: Na_8_Si_46_,^20^ Na_8_Ga_*x*_Si_46−*x*_,^12^ Sr_8_Ga_*x*_Si_46−*x*_,^8,9^ and Na_8−*y*_Sr_*y*_Ga_*x*_Si_46−*x*_ crystals 1 (Na_5.0_Sr_3.0_Ga_7.6_Si_38.4_), 2 (Na_4.2_Sr_3.8_Ga_8.4_Si_37.6_), and 3 (Na_3.9_Sr_4.1_Ga_9.1_Si_36.9_).

Fig. 4 shows the temperature dependence of electrical resistivity, *ρ*, measured for the three crystals and the type-I clathrate single crystal, Na_8_Ga_5.7_Si_40.3_, synthesized by our group in a previous study.^12^ The *ρ* values for crystals 1, 2, and 3 increased with increasing temperature, reaching 0.34, 0.55, and 1.05 mΩ cm, respectively, at room temperature (300 K). The previously reported electrical resistivities at 280–300 K for other type-I clathrates, Na_8_Si_46_,^20^ Na_8_Ga_*x*_Si_46−*x*_,^12^ and Sr_8_Ga_*x*_Si_46−*x*_,^8,9^ are compared to those in the Na_8−*y*_Sr_*y*_Ga_*x*_Si_46−*x*_ sample (crystal 1: Na_5.0_Sr_3.0_Ga_7.6_Si_38.4_, crystal 2: Na_4.2_Sr_3.8_Ga_8.4_Si_37.6_, and crystal 3: Na_3.9_Sr_4.1_Ga_9.1_Si_36.9_) with respect to their respective Ga contents, *x*, in Fig. 5. The electrical resistivity of crystal 1 was plotted at *x* = 7.6 in Fig. 5 even though one region in the Na_4.9(2)_Sr_3.3(2)_Ga_7.6(2)_Si_38.4(2)_ crystal had a composition of Na_8.3(2)_Ga_4.3(2)_Si_41.7(2)_ (as shown in Fig. S1†).

The resistivity of Na_8_Ga_*x*_Si_46−*x*_ was greater than that of Na_8_Si_46_ (0.098 mΩ cm) as measured previously by Stefanoski at 300 K ^20^ and increased with increasing *x*.^12^ Eight electrons are formally transferred from Na to the Si framework to form Na_8_Si_46_; these electrons enter into the conduction band. Since the valences of Si and Ga are 4 and 3, respectively, the number of the electrons (or carriers) in the conduction band should decrease with increasing Ga content in the [Si/Ga] framework, thus reducing the conductivities and changing the structure to a semiconducting Zintl clathrate with *x* = 8 like A_8_Ga_8_Si_38_ (A = K, Rb, Cs).^5^ The *ρ* values of Na_8−*y*_Sr_*y*_Ga_*x*_Si_46−*x*_ and Sr_8_Ga_*x*_Si_46−*x*_ followed trends similar to that shown in Fig. 5. Na_5.04_Sr_2.96(2)_Ga_7.6_Si_38.4_ (crystal 1), Na_4.20_Sr_3.80(3)_Ga_8.4_Si_37.6_ (crystal 2), and Na_3.92_Sr_4.08(2)_Ga_9.1_Si_36.9_ (crystal 3) have valence electron numbers of 3.36, 3.40, and 2.98, respectively, and crystal 3 exhibited the highest resistivity among Na_8−*y*_Sr_*y*_Ga_*x*_Si_46−*x*_. The resistivity of crystal 2 is higher than crystal 1 although the numbers are almost the same. The resistivity may also be related to the content of Ga atoms which scatter conduction carrier electrons and holes on the clathrate frame. The Ga content of crystal 2 (*x* = 8.40) is higher than crystal 1 (*x* = 7.6).

## 4. Summary

Here, single crystals of quaternary type-I clathrates, Na_8−*y*_Sr_*y*_Ga_*x*_Si_46−*x*_, were grown by heating a Na–Sr–Ga–Si melt at 773 K and evaporating the Na from the melt in an Ar atmosphere. The compositions and crystal structures of the single crystals were analyzed by EPMA and XRD. The Ga and Sr contents (*x* and *y*) of the obtained crystals were 7.6–9.1 and 2.96(2)–4.08(2), respectively. Na atoms preferentially occupied the Na/Si1(6d) sites, whereas Ga atoms preferentially occupied the Si/Ga3(6c) sites in the [Si/Ga]_24_ cages. The electrical resistivities of the Na_8−*y*_Sr_*y*_Ga_*x*_Si_46−*x*_ single crystals were found to increase from 0.34 to 1.05 mΩ cm with increasing *x* and *y* at 300 K.

## Conflicts of interest

There are no conflicts to declare.

## Supplementary Material

RA-009-C9RA01489F-s001
